# National population-based reference data for the Hip Disability and Osteoarthritis Outcome Score (HOOS)

**DOI:** 10.1007/s00402-023-04915-w

**Published:** 2023-06-06

**Authors:** Peter Larsen, Michael S. Rathleff, Ewa M. Roos, Rasmus Elsoe

**Affiliations:** 1 Department of Occupational Therapy and Physiotherapy, Aalborg University Hospital, Aalborg, Denmark; 2 Department of Sports Science and Clinical Biomechanics, University of Southern Denmark, Odense, Denmark; 3 Department of Orthopaedic Surgery, Aalborg University Hospital, Aalborg University, 18-22 Hobrovej, 9000 Aalborg, Denmark

**Keywords:** HOOS, HOOS-12, Hip, OA, Reference data, Normative data

## Abstract

**Introduction:**

Interpretation of patient-reported outcome scores such as the Hip Disability and Osteoarthritis Outcome Score (HOOS) can be improved with use of reference values. The aim of the study was to establish population-based reference values for the HOOS’ five subscales and its short-form HOOS-12.

**Materials and methods:**

A representative sample of 9997 Danish citizens 18 years and older were identified. The population record-based sample was based on seven predefined age groups and an equal sex distribution within each age group. A national secure electronic system was used to send the HOOS questionnaire and one supplementary question regarding previous hip complaints to all participants.

**Results:**

2277 participants completed the HOOS, 947 women (42%) and 1330 men (58%). The mean HOOS subscale scores were: pain 86.9 (95% CI 86.1–87.7), symptoms 83.7 (95% CI 82.9–84.5), ADL 88.2 (95% CI 87.5–89.0), sport and recreation function 83.1 (95% CI 82.0–84.1), QOL 82.7 (95% CI 81.8–83.6). The youngest age group reported better mean scores in four subscales compared to the oldest age group (pain 91.7 vs. 84.5, mean difference 7.2 95% CI 0.4–14.0), (ADL 94.6 points vs. 83.2, mean difference 11.4 95% CI 4.9–17.8), (sport and recreation function 91.5 points vs. 73.8 points, mean difference 17.7 95% CI 9.0–26.4), (QOL 88.9 points vs. 78.8, mean difference 10.1 points 95% CI 2.0–18.2). Participants with a self-reported hip complaint had worse HOOS scores across all subscales (mean difference range 22.1–34.6). Super obese patients (BMI > 40) had > 12.5 points worse scores across the five HOOS subscales. Results were similar for the HOOS-12.

**Conclusion:**

This study provides reference values for the HOOS and its short form HOOS-12. Results show that older patients and patients with a BMI over 40 have worse HOOS and HOOS-12 scores that may be of clinical importance in the interpretation of scores both when evaluating potential for improvement and post-treatment results.

## Introduction

During the last decades, patient-reported outcomes have become central in reporting the treatment effect and patients’ satisfaction following treatment of joint disorders [1]. Large-scale or national databases have been established in several countries worldwide, and patient-reported outcomes are commonly a central part of the registers to monitor quality of patient care [2].

In contrast to generic patient-reported measurement outcomes, body region-specific patient-reported outcome measurements can capture the patient perceived ability to perform specific activities indicatory for body regions such as the hip. This is often combined with questions covering health related quality of life^1^ [3]. In orthopedics, body region-specific patient-reported outcomes are used to capture the current status of the patient or as repeated questionnaires to describe the changes in a patients self-report status. These data are then often used in combination with radiological and functional measurements for clinical decision making.

The Hip Disability and Osteoarthritis Outcome Score (HOOS) is widely used body region-specific score used to investigate the effect following treatments of osteoarthritis and other hip related disabilities and is one of several internationally recommended patient-reported instruments to be used following hip replacement [4]. In 2019, a short form of HOOS named HOOS-12 was developed using item-response theory with the aim to reduce the respondent burden of patients especially in the clinical setting including scoring for joint replacement registries [5].

The interpretation of results from patient-reported outcome measurements such as the HOOS and HOOS-12 can be improved using large-scale normative data from randomly selected reference populations. Patient-reported outcomes scores are frequently reported to be influenced by age, sex, weight, social status, and comorbidity [6–10]. At present, reference data from the HOOS has been established from a single geographical region of southern Sweden and in a selected US population [7, 11]. No reference data are available for its short form HOOS-12. Accurate and large-scale reference data are essential to facilitate interpretation of HOOS and HOOS-12 scores and may be used as reference data for comparative study designs and in future power calculations.

The aim of the present study was to establish reference values for the Hip Disability and Osteoarthritis Outcome Score (HOOS) five subscales and its short form HOOS-12 based on a randomly selected population-based sample of adult Danes.

## Methods

### Study design

The study was designed as a population record-based sample, including a national representative sample of all citizens of Denmark.

At birth or emigration to Denmark, a Civil Registration Number (CPR) is given to all residents and registered in the Civil Registration System. The CPR number is mandatory by law for all citizens in Denmark. Prospective information regarding emigration and death is recorded in this registry [12]. The Civil Registration System includes individual information of the entire population of Denmark [12]. The CPR register was used to establish the study’s national and randomly selected sample of all Danish citizens. A secure digital mailing system (E-boks) is mandatory for almost all Danish citizens and is based on the Danish Civil Registration System.

The Danish Data Protection Agency approved the study (J. nr. 2021 Id: 114). The reporting of the study complies with the Strengthening the Reporting of Observational Studies in Epidemiology (STROBE) Statement [13].

### Data retrieval

Included were Danish citizens 18 years and older. Excluded were those without online contact information (E-boks). An invitation to participate in the project was mailed to all participants using the mandatory online system E-boks. The invitation included an online link to the HOOS questionnaire and contact information for the research group in case of questions. Before the study started, the invitation letter, HOOS, and the E-boks system were piloted on a small group of 10 participants. After finishing the HOOS questionnaire, participants were also asked to submit their height and weight. Furthermore, one supplemental question was asked: Within the last 5 years, have you been in contact with a health professional due to a hip problem? Answer: yes/no.

In case of no response within 14 days, participants received a second request to participate.

### Study population

A representative sample of 10,000 citizens of Denmark was derived from the Danish Civil Registration System. By 2021, the population of Denmark consisted of 5.8 million citizens.

The sample was selected based on seven predefined age groups (18–29, 30–39, 40–49, 50–59, 60–69, 70–79, 80 +) and an equal sex distribution across all groups. A sample of 10,000 citizens was defined and contacted to allow adequate power for subgroup analyses based on both age and gender. We expected a response rate of about 30% yielding approximately 200 citizens in each age and gender group.

### The Hip Disability and Osteoarthritis Outcome Score (HOOS) and its short form HOOS-12

The Hip Disability and Osteoarthritis Outcome Score (HOOS) is a patient-reported and body region-specific questionnaire that was introduced in 2003 with the aim of assessing patients’ experiences regarding their hip and associated problems [14]. It is a hip-specific, patient-administrated questionnaire which include 40 items across five subscales. These five subscales evaluate pain, symptoms, function of daily living (ADL), sport and recreation function (sport), and quality of life (QOL) [14]. The HOOS is available in more than 25 languages [14]. The HOOS is free to use and can be downloaded at www.koos.nu.

The short form HOOS-12 was developed using item response analysis and is calculated based on 12 items from the full HOOS questionnaire [5]. The outcome of HOOS-12 is reported in three subscales; pain, function and QOL, along with a aggregated overall hip impact summary score [5].

HOOS and HOOS-12 scores are calculated based on standardized scoring algorithms and presented as a score between 0 and 100 for each respective subscale [14]. A score of 100 indicates the best possible results and 0 the worst outcome [14].

The two scoring manuals are available at www.koos.nu.

### Statistical analysis

The HOOS and HOOS-12 scores are given as mean, median, standard deviation (SD), 95% confidence intervals (CI), minimum, maximum, and number for the overall group and for each age and gender group. In accordance with the HOOS scoring manual [14], scores were not calculated if the number of missing items was more than 50% in a subscale.

Continuous variables are reported by mean and standard deviation (SD) and categorical variables by frequencies. A two-way analysis of variance (ANOVA) was used to test the difference between predefined age and gender groups. If significant ANOVA factors or interactions were found, multiple pairwise analyses with post hoc test (Bonferroni) corrections were used.

One-way ANOVA was used to test the difference between subscales scores and reporting of hip problems (yes/no) and between subscales scores and BMI groups (18–24.9, 25–29.9, 30–34.9, 35–39.9, 40–44.9 and > 45), where BMI > 40 indicate the super obese. If a significant ANOVA factor was found, multiple pairwise analyses with post hoc test (Bonferroni) corrections were used.

Responders and non-responders were checked for age difference by the unpaired *t* test and for gender difference by the Chi-square test.

A *P* value of < 0.05 was considered significant. The statistical analysis was performed by STATA (version 27).

Thresholds suggested to represent a clinically relevant improvement range from 7 to 36 for the HOOS subscales, and from 15 to 28 for the HOOS-12 subscales [15–18]. In this paper, we considered thresholds of 8 for all HOOS subscales, and 15 for all HOOS-12 subscales and impact score, to represent a clinically relevant difference between age, sex and BMI groups.

## Results

**Fig. 1:**
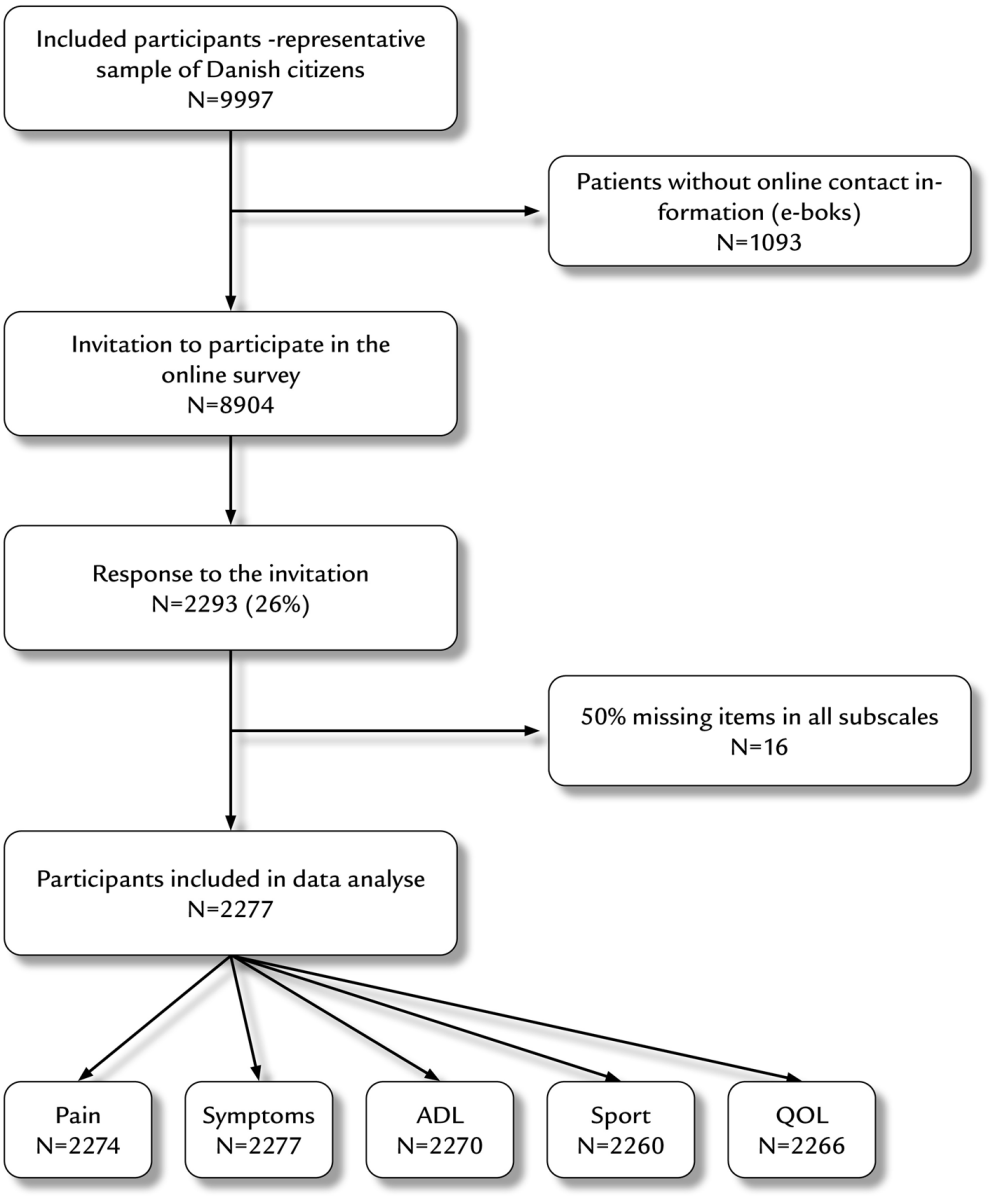
Detailed information regarding the full study population. *N = *number

Of the 9997 nationally representative persons identified, 1093 did not have a valid E-boks and could not be contacted. A total of 2277/8904 (26%) contacted participants completed the HOOS questionnaire, 947 women (41.6%) and 1330 men (58.4%). Detailed information regarding the full study population is presented in Fig. 1.

### HOOS and HOOS-12 reference scores

The HOOS and HOOS-12 subscale scores for the total sample and for the seven predefined age- and gender groups are presented in Tables 1 and 2, respectively.

**Table 1:** HOOS and HOOS-12 subscale scores for the total sample

	HOOS					HOOS12		
	Pain	Symptoms	ADL	Sport	QOL	Pain	Function	QOL
Mean	86.9	83.7	88.2	83.1	82.7	85.6	86.9	82.7
SD	19.2	19.8	18.2	24.9	22.9	20.4	19.7	22.9
95% CI	86.1–87.7	82.9–84.5	87.5–89.0	82.0–84.1	81.8–83.6	84.7–86.4	86.1–87.7	81.8–83.6
Median	97.5	90	98.5	100	93.8	100	100	93.8
Min	5	0	9	0	0	6	0	0
Max	100	100	100	100	100	100	100	100
Number	2274	2277	2270	2260	2266	2275	2268	2266

*SD* standard deviation, *CI* confidence interval

**Table 2:** HOOS and HOOS-12 subscale scores by age

HOOS Pain								HOOS Symptoms							
Age group	18–29	30–39	40–49	50–59	60–69	70–79	80 +	Age group	18–29	30–39	40–49	50–59	60–69	70–79	80 +
Mean	91.7	88.6	89.7	86.8	85.2	86.5	84.5	Mean	87.4	84.5	85.9	83.4	82.4	83.6	82.0
SD	17.3	18.3	17.1	20.4	19.9	18.8	19.0	SD	16.4	19.5	19.6	20.1	20.7	19.6	19.6
95% CI	88.4–95.0	86.1–91.1	87.7–91.7	84.8–88.8	83.5–86.9	84.8–88.1	82.0–87.0	95% CI	84.3–90.5	81.8–87.1	83.6–88.2	81.5–85.4	80.6–84.1	81.9–85.3	79.4–84.6
Median	100	100	100	100	98	98	93	Median	95	90	95	90	90	90	90
Min	10	5	15	18	13	18	18	Min	20	10	10	10	0	5	20
Max	100	100	100	100	100	100	100	Max	100	100	100	100	100	100	100
Number	109	210	282	407	538	505	223	Number	109	210	282	407	538	506	225

HOOS ADL								HOOS Sport							
Age group	18–29	30–39	40–49	50–59	60–69	70–79	80 +	Age group	18–29	30–39	40–49	50–59	60–69	70–79	80 +
Mean	94.6	91.2	92.0	89.0	86.5	86.8	83.2	Mean	91.5	88.3	89.1	85.2	81.5	79.6	73.8
SD	13.7	16.4	15.2	18.2	19.2	18.4	20.1	SD	18.0	21.0	19.0	24.1	25.6	26.8	28.6
95% CI	92.0–97.2	89.0–93.4	90.3–93.8	87.2–90.7	84.9–88.1	85.2–88.4	80.6–85.9	95% CI	88.1–94.9	85.4–91.2	86.9–91.3	82.8–87.5	79.4–83.7	77.2–81.9	70.0–77.6
Median	100	100	100	99	97	96	93	Median	100	100	100	100	100	94	88
Min	22	24	10	24	9	15	22	Min	0	0	6	0	0	0	0
Max	100	100	100	100	100	100	100	Max	100	100	100	100	100	100	100
Number	109	210	282	407	537	504	221	Number	109	210	281	406	535	498	221

HOOS QOL								HOOS12 Pain							
Age group	18–29	30–39	40–49	50–59	60–69	70–79	80 +	Age group	18–29	30–39	40–49	50–59	60–69	70–79	80 +
Mean	88.9	85.3	85.2	83.6	80.4	82.3	78.8	Mean	90.8	87.9	88.3	85.7	83.6	85.4	83.4
SD	19.5	21.2	22.2	22.9	23.8	22.1	24.8	SD	17.4	19.3	18.6	21.6	21.3	19.8	20.7
95% CI	85.2–92.6	82.4–88.1	82.6–87.8	81.4–85.8	78.4–82.4	80.4–84.3	75.6–82.1	95% CI	87.4–94.1	85.3–90.5	86.2–90.5	83.6–87.8	81.8–85.4	83.6–87.1	80.7–86.2
Median	100	100	100	100	94	94	88	Median	100	100	100	100	94	94	94
Min	6	19	13	0	0	13	13	Min	6	6	13	13	13	19	6
Max	100	100	100	100	100	100	100	Max	100	100	100	100	100	100	100
Number	109	210	281	407	536	502	221	Number	109	210	282	407	538	505	224

HOOS12 Function								HOOS12 QOL							
Age group	18–29	30–39	40–49	50–59	60–69	70–79	80 +	Age group	18–29	30–39	40–49	50–59	60–69	70–79	80 +
Mean	94.3	90.5	91.4	87.4	85.3	85.2	81.4	Mean	88.9	85.3	85.2	83.6	80.4	82.3	78.8
SD	14.2	17.4	16.3	20.0	20.8	20.0	21.4	SD	19.5	21.2	22.2	22.9	23.8	22.1	24.8
95% CI	91.6–97.0	88.1–92.8	89.5	85.5–89.4	83.5–87.1	83.5–87.0	78.6–84.3	95% CI	85.2–92.6	82.4–88.1	82.6–87.8	81.4–85.8	78.4–82.4	80.4–84.3	75.6–82.1
Median	100	100	93.3	100	94	94	88	Median	100	100	100	100	94	94	88
Min	13	19	13	6	0	13	13	Min	6	19	13	0	0	13	13
Max	100	100	100	100	100	100	100	Max	100	100	100	100	100	100	100
Number	109	210	282	406	537	504	220	Number	109	210	281	407	536	502	221

*SD* standard deviation, *CI *confidence interval

### Age- and gender specific scores for the HOOS and HOOS-12

**Fig. 2:**
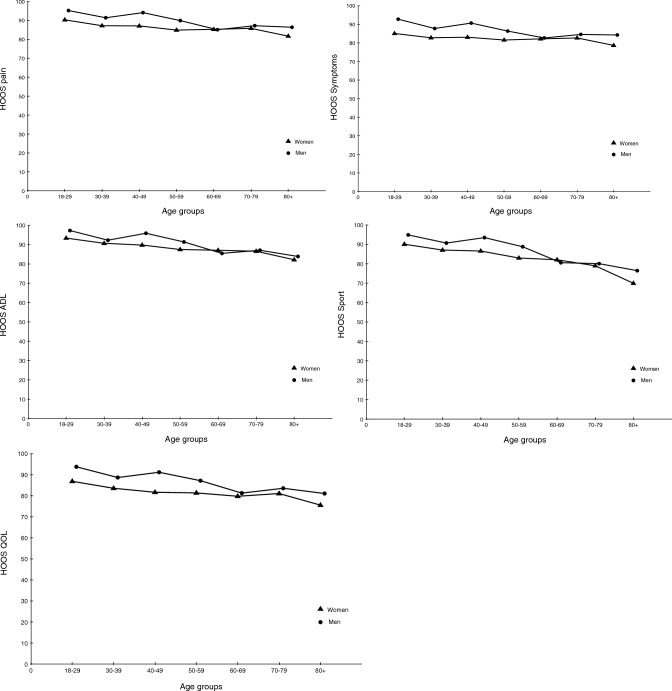
HOOS subscales divided by age groups and gender

The age- and gender specific HOOS and HOOS-12 subscale scores are presented in Fig. 2 and Table 2, respectively. Women reported statistically significant worse scores for three of the five HOOS subscales (pain mean difference 2.6, 95% CI 0.9–4.2), (symptoms mean difference 3.3 95% CI 1.7–5.0), (QOL mean difference 3.7 95% CI 1.8–5.6) and for the HOOS-12 subscale scores (pain mean difference 2.9 95% CI 1.2–4.6), (QOL mean difference 3.5 95% CI 1.8–5.6) and for the HOOS-12 impact score (mean difference 2.6 95% CI 0.9–4.3).

Comparing the youngest and oldest age groups a significant difference were observed in four of the five HOOS subscales (pain mean difference 7.2 95% CI 0.4–14.0), (ADL mean difference 11.4 95% CI 4.9–17.8), (sport and recreation function mean difference 17.7 95% CI 9.0–26.4), (QOL mean difference 10.1 points 95% CI 2.0–18.2) and for the HOOS-12 (pain mean difference 7.3 95% CI 0.1–14.5), (ADL mean difference 12.9 95% CI 6.0–19.8), (QOL mean difference 10.1 95% CI 2.0–18.1) and (impact score mean difference 10.2 95% CI 3.1–17.4).

Excluding patients reporting a hip complaint reduced the difference between age groups in three of the five HOOS (ADL mean difference 8.6 95% CI 3.1–14.1), (sport and recreation function mean difference 13.6 95% CI 5.7–21.5), (QOL mean difference 8.2 points 95% CI 1.4–15.1.

### Impact of hip complaint within 5 years on HOOS/HOOS-12 scores

A total of 377 participants (17%) reported a hip complaint within the last 5 years. Of the 377 participants reporting a hip complaint, most were 50 years of age or older (75%), and most were women (67%). Reporting a hip complaint within the past 5 years was associated with worse HOOS scores compared to participants without reporting hip complaints: (pain mean difference 25.4 95% CI 23.6–27.3), (symptoms mean difference 25.2 95% CI 23.3–27.1), (ADL mean difference 22.1 95% CI 20.3–23.9), (sport mean difference 29.0 95% CI 26.5–31.5) and (QOL mean difference 34.6 95% CI 32.5–36.7). Similar differences between groups were found when using the HOOS-12 subscales and the impact score: (pain mean difference 27.3 95% CI 25.4–29.3), (function mean difference 23.3 95% CI 21.3–25.2), (QOL mean difference 34.6 95% CI 32.5–36.7) and (impact score mean difference 28.4 95% CI 26.1–30.8).

### Impact of BMI on HOOS and HOOS-12 scores

The mean self-reported BMI was 26.8 (± 11.5). Nineteen percent (*N = *428) of participants reported a BMI of 30 or higher. Markedly worse scores were found in participants reporting a BMI over 40, Table 3.

**Table 3:** HOOS and HOOS-12 subscale scores by BMI groups

HOOS Pain					HOOS Symptoms					HOOS ADL				
BMI-groups	Mean	95% CI	SD	Number	BMI-groups	Mean	95% CI	SD	Number	BMI-groups	Mean	95% CI	SD	Number
18–24.9	90.6	89.6–91.7	16.4	960	18–24.9	87.0	85.9–88.1	17.8	961	18–24.9	91.8	90.8–92.8	15.8	959
25–29.9	84.5	83.1–86.0	20.8	790	25–29.9	82.0	80.5–83.5	21.3	791	25–29.9	86.4	85.0–87.7	19.3	790
30–34.9	84.5	82.2–86.8	19.3	278	30–34.9	80.8	78.4–82.9	19.0	278	30–34.9	85.4	83.3–87.6	18.3	277
35–39.9	82.7	78.5–86.9	20.8	96	35–39.9	78.4	73.8–83.0	22.7	96	35–39.9	83.5	79.3–87.6	20.7	96
40–44.9	77.0	68.4–85.5	22.1	28	40–44.9	75.2	66.3–84.1	23.0	28	40–44.9	78.9	70.2–87.6	22.4	28
over 45	76.1	67.1–85.0	22.2	26	over 45	73.1	64.0–82.2	22.5	26	over 45	78.7	69.8–87.7	22.2	26

HOOS Sport					HOOS QOL					HOOS12 Pain				
BMI-groups	Mean	95% CI	SD	Number	BMI-groups	Mean	95% CI	SD	Number	BMI-groups	Mean	95% CI	SD	Number
18–24.9	88.2	86.9–89.5	21.1	956	18–24.9	86.3	85.0–87.7	21.1	958	18–24.9	89.6	88.5–90.7	17.7	960
25–29.9	80.5	78.6–82.4	26.6	786	25–29.9	80.1	78.4–81.8	24.3	787	25–29.9	83.3	81.7–84.8	22.1	790
30–34.9	78.7	75.6–81.8	26.0	276	30–34.9	80.2	77.6–82.9	22.4	277	30–34.9	83.1	80.7–85.4	20.3	278
35–39.9	75.1	69.5–80.6	27.3	95	35–39.9	80.0	75.5–84.5	22.1	96	35–39.9	81.8	77.3–86.3	22.2	96
40–44.9	70.8	28.1–83.4	32.6	28	40–44.9	74.1	63.6–84.6	27.1	28	40–44.9	74.3	64.4–84.1	25.4	28
over 45	74.5	62.8–86.3	29.1	26	over 45	73.8	63.4–84.2	25.7	26	over 45	74.4	65.5–83.3	22.0	26

HOOS12 Function					HOOS12 QOL				
BMI-Groups	Mean	95% CI	SD	Number	BMI-groups	Mean	95% CI	SD	Number
18–24.9	90.9	89.8–92.0	16.7	958	18–24.9	86.3	85.0–87.7	21.1	958
25–29.9	84.8	83.3–86.2	21.1	790	25–29.9	80.1	78.4–81.8	24.3	787
30–34.9	84.3	81.9–86.6	20.2	277	30–34.9	80.2	77.6–82.9	22.4	277
35–39.9	80.7	75.9–85.4	23.4	96	35–39.9	80.0	75.5–84.5	22.1	96
40–44.9	77.5	68.1–86.9	24.3	28	40–44.9	74.1	63.6–84.6	27.1	28
over 45	78.6	70.0–87.2	21.2	26	over 45	73.8	63.4–84.2	25.7	26

*BMI* body mass index, *SD* standard deviation, *CI* confidence interval

## Discussion

This study is the first to report HOOS and HOOS-12 reference values from a nationally representative sample. We report reference data across age groups from 18 years of age and older in 10-year strata and subdivided into men and women and the impact of BMI. These reference values will improve the interpretation of HOOS and HOOS-12 collected in clinical practice and research.

### HOOS/HOOS-12 and age

This study indicated that the use of age-specific HOOS scores could be warranted. We found statistically significant worse HOOS/HOOS-12 scores with increasing age. Our findings are in line with other studies reporting worse HOOS scores with increasing age[7, 11, 19]. This supports the use of age-strata when interpreting outcomes from clinical practice across age groups. The impact of age differences may affect how the reference values are used in clinical practice and in research. For an individual patient, the appropriate age-strata would be useful, as expected treatment results are likely not to increase above the level of age-specific HOOS reference scores. For use in research where the specific patient-population spans several age-strata it may be useful to check the difference in reference values across the relevant age-strata, and consider if this difference is of clinical relevance, or not. Prevalence of hip complaints increase with older age, indicating that the effect of hip complains are imbedded in the age-related differences in the HOOS subscale scores. However, when removing participants with hip complaints the difference between the youngest and oldest age group decrease 2–4 points across the different subscales.

### HOOS/HOOS-12 and sex

This study indicates that the use of sex specific HOOS subscales is not needed. In this study we found significantly worse HOOS and HOOS-12 outcomes on three of the five HOOS subscales for women compared to men. Although statistically significant differences, the current body of knowledge do not indicate these differences of 2.6–3.7 points are clinically important[15, 16, 18, 20]. Findings were similar across the 7 predefined age groups.

### HOOS/HOOS-12 and BMI

We found that being super obese (BMI > 40) was associated with substantially worse HOOS and HOOS-12 scores. The difference between normal weight and the super obese were > 12 points on the HOOS/HOOS-12 subscales indicating likely clinically relevant differences between groups that needs to be considered when using HOOS scores for interpreting patient outcomes. These results are in line with Raja et al. [11] who reported a comparable association between higher BMI and worse HOOS score in a selected US population. Reference data for the super obsese may assist in guiding health care workers and patients when considering the expected level of HOOS and HOOS-12 scores following treatment for a hip problem.

### Use of HOOS reference data in clinical and research settings

The use of HOOS reference data in the orthopedic setting is twofold. When used in the clinic for evaluation of a specific treatment, reference data may help indicate the expected improvement and provide a treatment goal. Age specific reference values may be of particular relevance when for example treating an older woman with a hip fracture, and BMI-specific reference values may be of particular relevance to indicate the expected improvement for a superobese or obese patient having total hip replacement. Reference values are also usable when applied to groups of patients in orthopedic research. When HOOS or HOOS-12 is considered as the primary outcome in a future study, reference values support the initial planning of the study by indicating optimal mean scores following treatment.

### Strengths and limitations

A strength of this study is the randomly selected national age and gender representative sampling base, with more than 2200 responders making this study the largest HOOS/HOOS-12 reference material available. Missing responses were spread across age and gender subgroups minimizing the risk of a strong selection bias. Responders and non-responders did not differ on age and gender, it is not possible to test if non-responders were different on other variables.

## Conclusion

This study is based on a national randomly selected population and provides sex and age-specific reference values of the Hip Disability and Osteoarthritis Outcome Score (HOOS) and its short form HOOS-12. We found that older patients and patients with a BMI more than 40 have clinically relevant worse HOOS and HOOS-12 scores. Using age- and BMI-specific reference values may therefore be indicated when evaluating potential for improvement and post-treatment results in these patient groups.

## Data Availability

The datasets generated and analyzed during the current study are in part available from the corresponding author on reasonable request.

## Footnotes

1 © 2015 Research Triangle Institute. All rights reserved. This book is protected by copyright. Credit must be provided to the author and source of the book when the content is quoted. No part of this book may be reproduced in any form by any electronic or mechanical means (including photocopying, recording, or information storage and retrieval) without permission in writing from the publisher., Research Triangle Park (NC).
